# Upon the Pian or Yaws, a Disease of Tropical Climates

**Published:** 1853-11

**Authors:** 


*Upon the Pian or Yaws, a Disease of Tropical Climates.* By M. GUYON.—This eruptive disease, called, from the raspberry-like color of the spots, frambæsia, appears to be contagious, and transmissible from generation to generation. Its transmissibility by inoculation is proved by the fact, that it has been conveyed from the tropical regions of America to Africa, doubtless by infected negroes landing from ships trading between the two countries. In Algeria, the native physicians consider it to be a sort of syphilis, and treat it as such. They direct, for forty days, a strict system of diet, under which the disease disappears, but it returns as soon as the patient reverts to his usual habits. From this fact, M. Guyon concludes, that the apparent cure is but a phenomenon connected with the general emaciation and disappearance of the scft parts.

In the Antilles, the treatment of yaws is entrusted to negroes, the same class of men who profess to cure serpent bites. They administer decoction of sarsaparilla and sulphate of ammonia. Some physicians, it is said, have tried with good effect mercurials combined with tisanes of guaiacum and sarsaparilla. The usual duration of the disease is four to five months. We require further investigations respecting it.—*London Medical Times.*

